# Impact of APOE3/E3 Astrocytes on APOE4/E4 Neuronal Phenotype

**DOI:** 10.1002/alz70855_106137

**Published:** 2025-12-24

**Authors:** Shuhan Li

**Affiliations:** ^1^ University of Oxford, Oxford, OXFORD, United Kingdom

## Abstract

**Background:**

Introduction: Alzheimer's disease (AD) is the most common form of dementia with APOE presenting the highest genetic risk factor (AD risk: APOE2 < APOE3 < APOE4 allele) and is predominantly expressed by astrocytes. Neuron‐astrocyte interactions have been proposed to alleviate certain pathophysiological processes in AD, including Ca2+ regulation, synaptic transport, glycogen metabolism etc. However, the pathways that mediate these interactions are unclear and it is unknown if they are impacted by the APOE genotype of either the astrocyte or the neuron. This study investigates the ability of isogenic induced pluripotent stem cells (iPSCs) ‐ derived astrocytes to affect the phenotype of iPSCs‐neurons carrying APOE3/E3 (E3) or APOE4/E4 (E4) backgrounds using both conditioned media (ACM) and co‐culture to identify any requirements for direct cell contact.

**Method:**

Immunocytochemistry was performed to track cellular morphology and organelle changes using markers including MAP2 (neuronal skeleton), EEA1 (early endosomes), S100B (astrocytes skeleton), BODIPY (neutral lipids), SYP (pre‐synaptic proteins) and DAPI (nuclei). Feature extraction was performed using Harmony (PerkinElmer) analysis, followed by traditional high‐content image analysis to quantify cellular changes and features of interest. Machine learning (ML) was applied to classify genotypes and assess similarities between groups based on these features. Random forest analysis was used to identify the most important features contributing to the decision‐making process.

**Result:**

Isogenic E3 or E4 neurons can be correctly classified using ML from both ACM and coculture groups. Specifically, neurite segment length, EEA1 intensity and roundness, number of SYP per neuron were identified as important features in understanding the effects of ACM on neurons. E4/4 neurons have higher EEA1 intensity and roundness across ACM groups. Neuronal EEA1 number and size, number of neurons were identified as important features in understanding the effects of astrocyte co‐culture.

**Conclusion:**

Nneurons with the same APOE genotype are more similar despite the genotypes of the treated ACM. The difference in response of neurons to ACM treatment suggests a potential donor effect, warranting further investigation. Yet, the morphology of neurons is affected by the co‐cultured astrocytes, suggesting that the influence of co‐cultured astrocytes is more salient that the neurons’ genotype.

